# Comparison of Lysis and Detachment Sample Preparation Methods for Cultured Triple-Negative Breast Cancer Cells Using UHPLC–HRMS-Based Metabolomics

**DOI:** 10.3390/metabo12020168

**Published:** 2022-02-10

**Authors:** Blake R. Rushing, Madison Schroder, Susan C. J. Sumner

**Affiliations:** Department of Nutrition, Nutrition Research Institute, University of North Carolina-Chapel Hill, Kannapolis, NC 28081, USA; schrodm@email.unc.edu (M.S.); susan_sumner@unc.edu (S.C.J.S.)

**Keywords:** cell metabolomics, mass spectrometry, breast cancer, pathway analysis, detachment, lysis

## Abstract

Dysregulation of cellular metabolism is now a well-recognized hallmark of cancer. Studies investigating the metabolic features of cancer cells have shed new light onto processes in cancer cell biology and have identified many potential novel treatment options. The advancement of mass spectrometry-based metabolomics has improved the ability to monitor multiple metabolic pathways simultaneously in various experimental settings. However, questions still remain as to how certain steps in the metabolite extraction process affect the metabolic profiles of cancer cells. Here, we use ultra-high-performance liquid chromatography–high-resolution mass spectrometry (UHPLC–HRMS) untargeted metabolomics to investigate the effects of different detachment and lysis methods on the types and abundances of metabolites extracted from MDA-MB-231 cells through the use of in-house standards libraries and pathway analysis software. Results indicate that detachment methods (trypsinization vs. scraping) had the greatest effect on metabolic profiles whereas lysis methods (homogenizer beads vs. freeze–thaw cycling) had a lesser, though still significant, effect. No singular method was clearly superior over others, with certain metabolite classes giving higher abundances or lower variation for each detachment–lysis combination. These results indicate the importance of carefully selecting sample preparation methods for cell-based metabolomics to optimize the extraction performance for certain compound classes.

## 1. Introduction

Metabolic reprogramming has emerged as a hallmark of cancer, and is known to play a critical role in a multitude of biological processes, including drug resistance, activating metastasis, responding to elevated oxidative stress, adapting to hypoxic conditions, protecting against tumor-infiltrating immune cells, inducing angiogenesis, and other processes that are necessary for the survival, growth, and spread of tumors in the body [1,2,3]. This has led to a resurgence of interest in cancer cell metabolism to understand cancer biology and potential preventative or therapeutic options. Furthermore, it has become clear that many aspects of cancer cell metabolism can, in part, be under oncogenic control, forming a link between genetic mutations, gene expression, epigenetic alterations, and metabolism [4,5]. As such, studying metabolic processes in cancer research can be a powerful approach to gain a deeper understanding of the biological processes and potential therapeutic targets relevant to this disease.

For these reasons, metabolomics has become a powerful tool in cancer research. Advancements in mass spectrometry has enabled the detection of tens of thousands of spectral features in biological specimens, due to increases in sensitivity and chromatographic resolution [6]. Furthermore, the increased stability of mass accuracy and more rigorous metabolite reporting standards have allowed for the identification of more metabolites with increased confidence [7]. Using metabolomics to investigate the metabolic underpinnings of cancer-related cellular mechanisms and the response to front-line treatment and new intervention strategies has increased due to the ability to simultaneously examine large numbers of pathways and metabolic drivers [8]. Challenges that remain include considerable variability in metabolic signatures that can arise from procedures used in extracting metabolites from cultured systems. Investigations have shown that methods for quenching, detachment, and cell lysis can have significant effects on the type and abundance of metabolites that can be analyzed with downstream instrumentation [9,10,11,12,13,14,15,16]. These effects can be cell line dependent, which further necessitates the need to investigate how sample preparation procedures affect extractable metabolites in different culture models [10,17].

**Figure 1:**
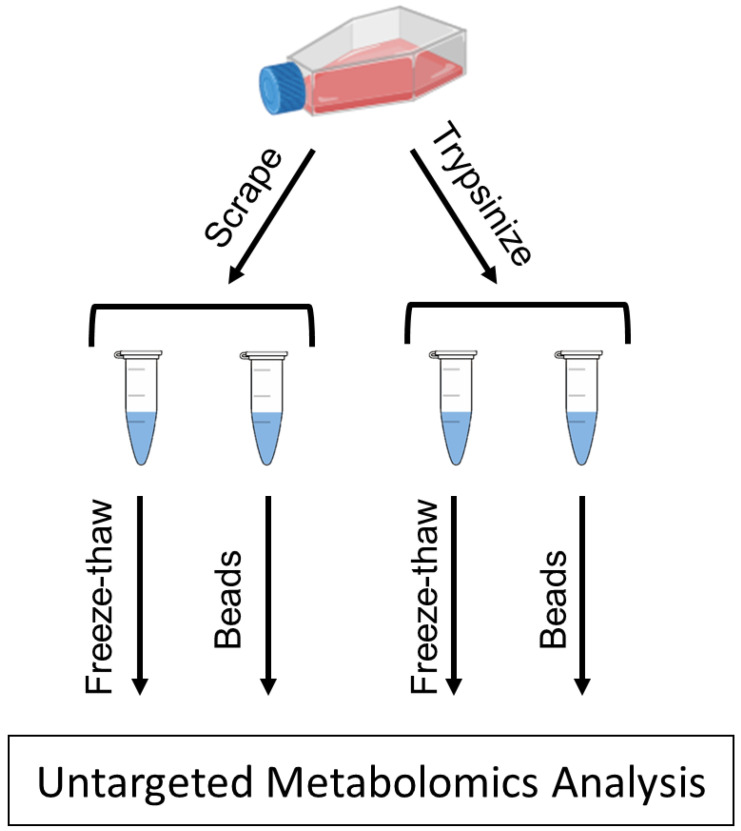
**Sample preparation workflow.** Adherent MDA-MB-231 cells were detached from flasks by either scraping or the use of trypsin. After each detachment method, cells were lysed by freeze–thaw cycling or by homogenizer beads. Cell lysates were then analyzed by UHPLC–HRMS to determine differences in metabolite abundances between the different methods.

In this study, we present the effects of two detachment methods—trypsinization and cell scraping—as well as two physical lysis methods—freeze–thaw cycling and bead homogenization—on the types and abundances of metabolites extracted from MDA-MB-231 cells analyzed by ultra-high-performance liquid chromatography–high-resolution mass spectrometry (UHPLC–HRMS) (Figure 1). The MDA-MB-231 cell line is commonly used as a model of triple-negative breast cancer [18]. Using an in-house physical standards library, we describe the endogenous metabolites that were detected for the detachment and lysis methods, and present compounds belonging to a diverse set of metabolite classes/pathways in MDA-MB-231 cells. We also describe how these detachment and lysis methods affect the levels of different metabolite classes and pathways. This information will aid in allowing metabolomics researchers to choose the most appropriate sample preparation procedures for studies using this cell model, and to consider the effects of some of these processes on the metabotypes of the cells at the time of extraction.

## 2. Results

**Figure 2:**
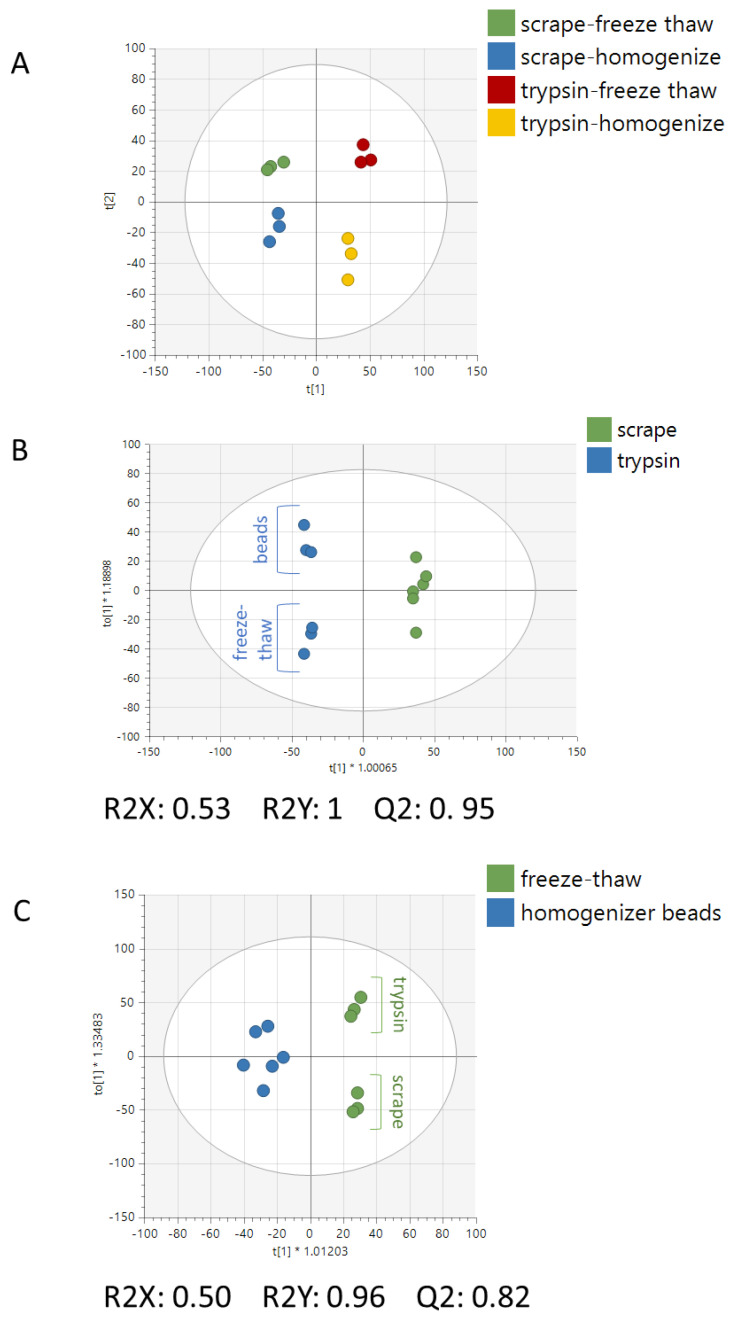
**Multivariate analysis of the extracted cell samples.** (**A**) PCA of all samples showing separation of different detachment and lysis methods. (**B**) OPLS-DA of all trypsinized samples versus all cell-scraped samples. Sub-splitting can be seen between the bead and freeze–thaw lysis methods, particularly in the trypsinized samples. (**C**) OPLS-DA of all freeze–thawed samples versus all bead-homogenized samples. Sub-splitting can be seen between the bead and freeze–thaw lysis methods, particularly in the freeze–thawed samples.

Following normalization and preprocessing, 4479 peaks remained in the metabolomics dataset. A clear distinction between all four extraction methods can be visualized in the PCA scatter plot (Figure 2A). Supervised analyses were built to evaluate the model statistics for differentiation of the metabolite profiles for cells derived from the detachment methods or lysis methods. Model statistics indicated that both the detachment method (Figure 2B) and lysis method (Figure 2C) produced strong differences in metabolomes, as seen by the high values for R2X and R2Y and Q2 values > 0.5—a benchmark that is widely accepted for metabolomics data as an indication of reproducibility [19]. These results indicate that both detachment methods and both lysis methods each produce a distinct metabolic profile from one another.

**Figure 3:**
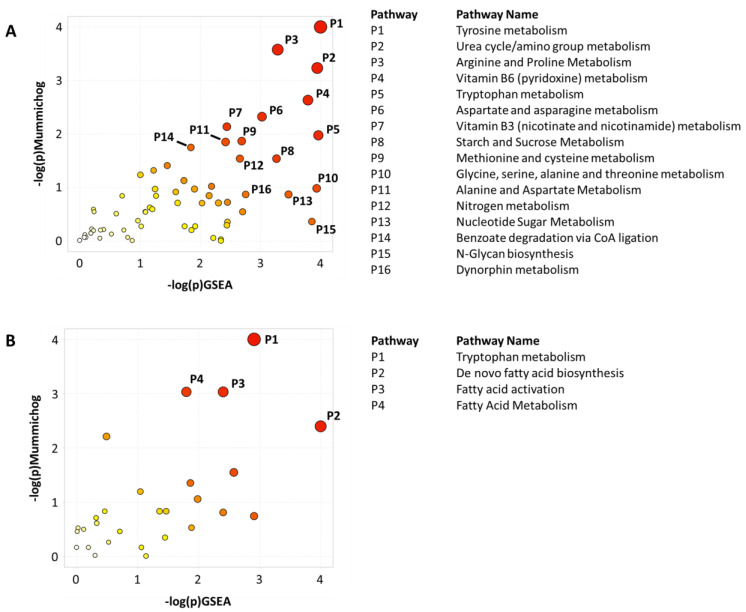
**Metabolic pathways impacted by sample preparation methods.** Pathway analysis was performed between (**A**) trypsinized vs scraped samples, and (**B**) freeze–thaw vs. homogenizer bead samples using MetaboAnalyst. Both the mummichog and GSEA algorithms were performed to determine the enriched pathways. Pathways with an integrated *p*-value less than 0.05 are annotated. Pathways are numbered in order of increasing integrated *p*-value.

Pathway analysis was performed to identify the biochemical pathways that were significantly different between the detachment and lysis methods. Peak intensity tables were imported into MetaboAnalyst’s “Functional Analysis” module to compare the trypsinized vs. scraped samples as well as homogenizer beads vs freeze–thaw cycling lysis methods. Results indicated that the detachment method (Figure 3A) perturbed a larger number of metabolic pathways as compared to the lysis method (Figure 3B). Sixteen metabolic pathways were significantly altered between the trypsinized and scraped samples and represented many different metabolite classes, such as amino acids, vitamins, sugars, and nucleotides. In contrast, the comparison between samples lysed by homogenizer beads and samples lysed by freeze–thaw cycling showed metabolic perturbations primarily in processes related to fatty acids. Pathway *p*-values for both the mummichog and GSEA algorithms, as well as the combined *p*-value, are given in Table 1 for pathways with a combined *p*-value < 0.05.

**Figure 4:**
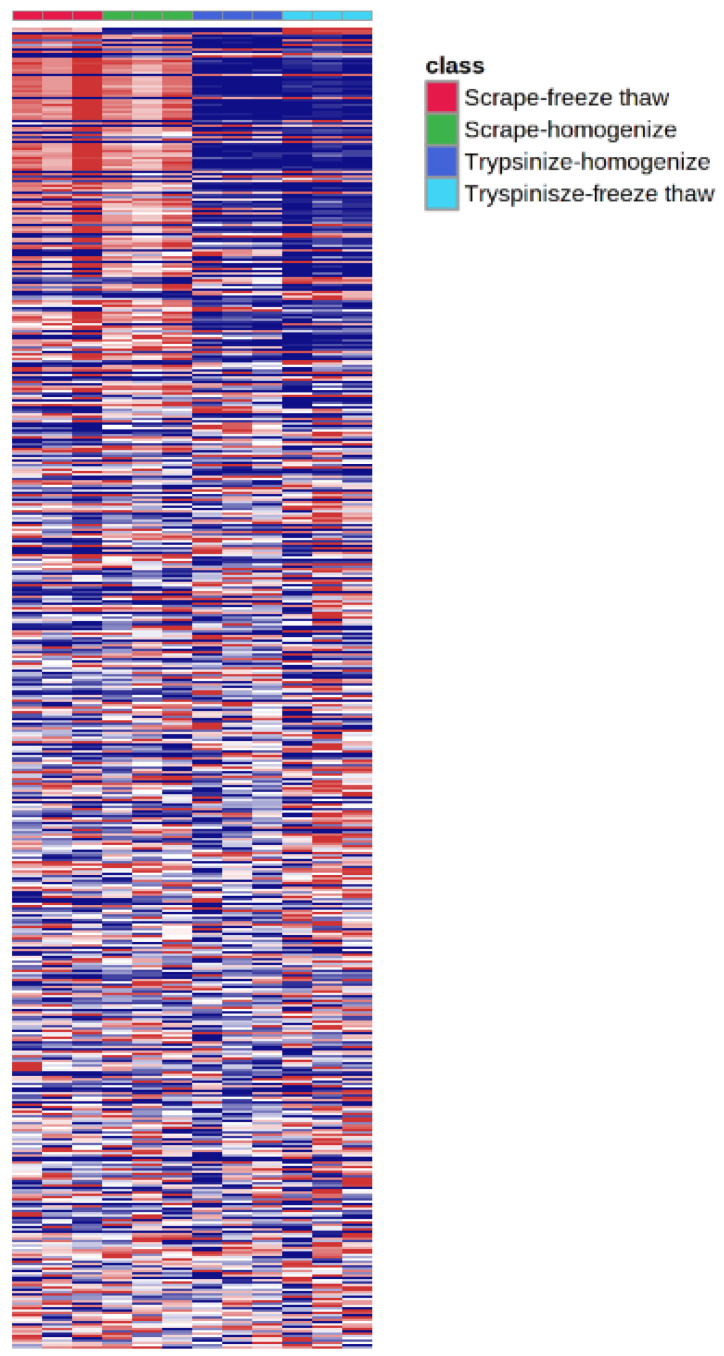
**Heatmap of all metabolomics peaks.** All peaks were imported into MetaboAnalyst to visualize the peak abundances across different sample preparation methods. The detachment method showed the largest difference in metabolome profiles as compared to the lysis method.

**Figure 5:**
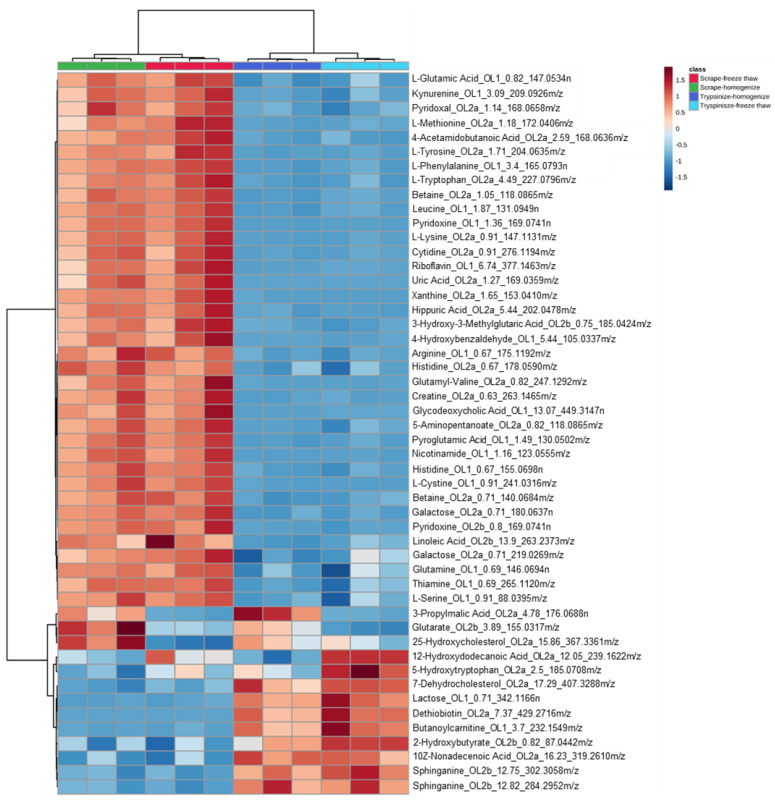
**Heatmap of the top 50 metabolites by ANOVA *p*-value.** Signals matched to the in-house library were input into MetaboAnalyst’s “Statistical Analysis” module to generate a heatmap across the different sample preparation methods. Metabolites are displayed in the following format: compound name_ontology level_retention time_mass. Masses ending with *n* are neutral masses whereas masses ending in *m*/*z* are ion masses.

**Figure 6:**
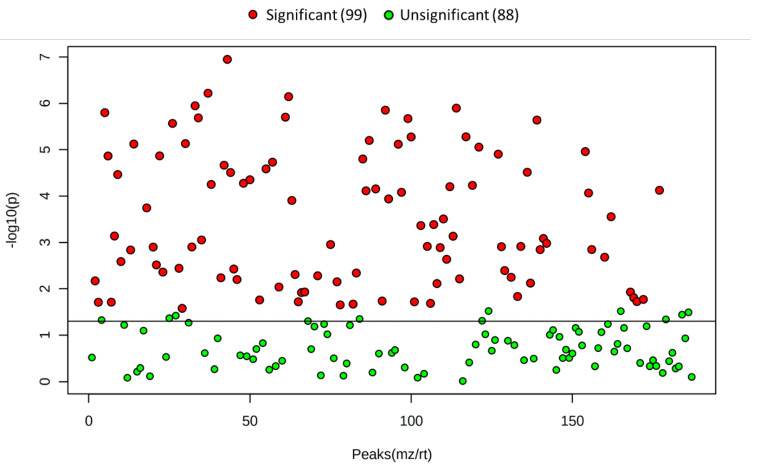
ANOVA scatter plot of the identified metabolites across each extraction method. Each point on the scatter plot represents one signal that matched to the in-house library of standards.

**Figure 7:**
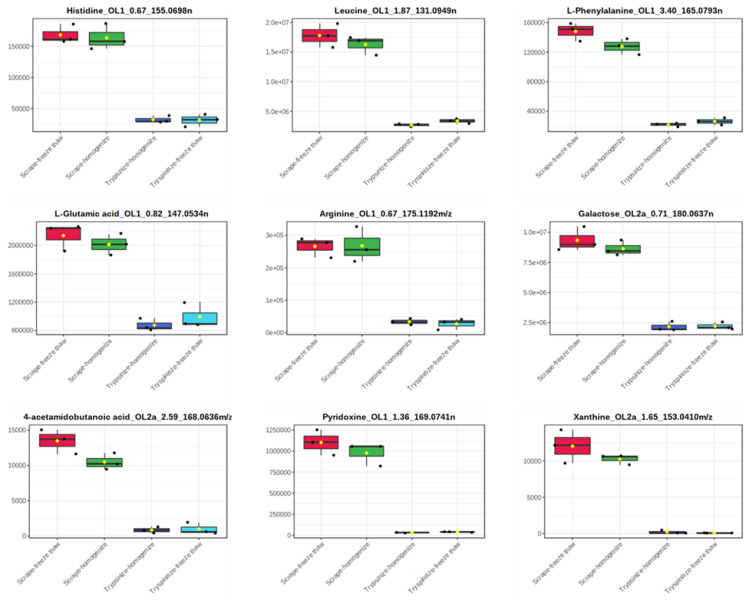
Representative metabolites that were increased in the scraped samples. Metabolites are displayed in the following format: compound name_ontology level_retention time_mass. Masses ending with *n* are neutral masses whereas masses ending in *m*/*z* are ion masses.

**Figure 8:**
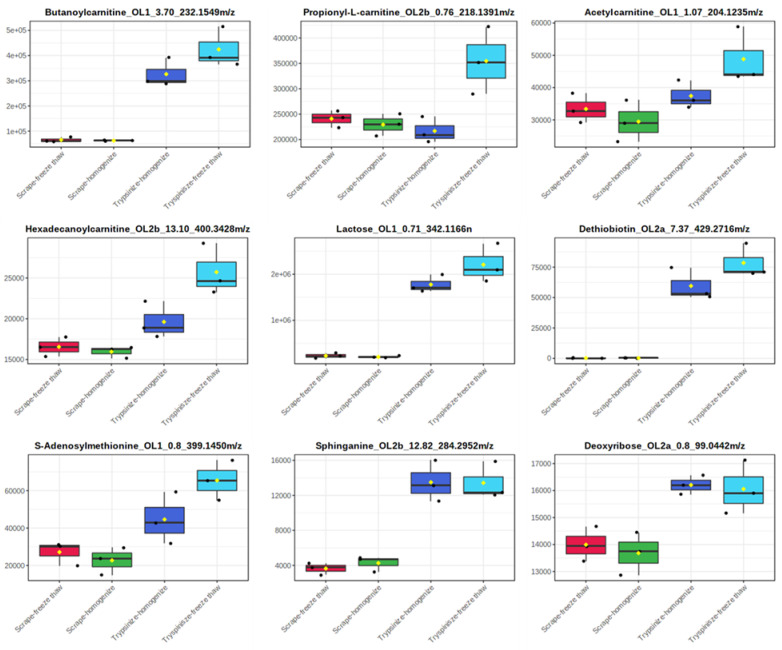
Representative metabolites that were increased in trypsinized samples. Metabolites are displayed in the following format: compound name_ontology level_retention time_mass. Masses ending with *n* are neutral masses whereas masses ending in *m*/*z* are ion masses.

While the majority of peaks were higher in the scraped samples, many signals were instead higher in the trypsinized samples. In general, good reproducibility was seen among the different sample preparation methods for each peak (Figure 4). A heatmap of the top 50 identified metabolites by ANOVA *p*-value also followed this trend (Figure 5). These top 50 metabolites represent many of the significant metabolic pathways identified in the pathway analysis. In total, 99 out of the 187 metabolites identified using the in-house library at the OL1, OL2a, or OL2b ontology level had an ANOVA *p*-value < 0.05 across the different extraction methods (Figure 6), indicating that the levels of these metabolites were significantly affected by the different sample preparation methods used in this study. Examples of metabolites with higher abundances in scraped samples include amino acids (such as histidine, leucine, phenylalanine, and glutamic acid) as well as other metabolites related to vitamin metabolism and the urea cycle (representative compounds shown in Figure 7). In contrast, trypsinized samples had higher levels of lactate, acylcarnitines, and other fatty acid-related metabolites (representative compounds shown in Figure 8). In general, the two lysis methods gave similar metabolite levels, although some acylcarnitines in the trypsinized samples were noticeably higher in the freeze–thawed samples as compared to those lysed by homogenizer beads. Table 2 provides a list of the average abundance and relative standard deviation (RSD) of all metabolites across all four methods. In terms of reproducibility, no singular method seemed to be superior to the others. Rather, each method has certain compounds with low RSD and high RSD values. Table S1 displays the variation of each named metabolite across the quality control study pool (QCSP) injections. Greater than 90% of the named metabolites had an RSD less than 30% across the QCSP injections, indicating sufficient analytical reproducibility for this study.

## 3. Discussion

In the current study, we set out to investigate how different sample preparation methods affected the types and abundances of metabolites that could be extracted from MDA-MB-231 cells for metabolomics analysis. Two major steps of cell sample preparation were investigated: detachment and lysis methods. Overall, it was observed that the detachment method had the greatest effect on peak abundance and affected a larger number of signals than the lysis methods. Overall, scraped samples had higher peak abundances than trypsinized samples. This observation has been made in other studies using other cell types [10,11,16,17,20]. This phenomenon of trypsinization causing a decrease in metabolite abundance has been referred to as “metabolite leakage” and it is thought that trypsin leads to permeabilization of cell membranes, allowing metabolites to leak out of the cell and into the extracellular space [20]. However, it should be noted that a large number of signals were increased in trypsinized samples, which were matched to metabolites related to fatty acid metabolism, such as acylcarnitines. This observation suggests that many of these metabolic differences may arise due to a biological response to the trypsinization process. Typically, cells are incubated with trypsin for ~5–10 min in order to sufficiently degrade extracellular matrix proteins and complete the detachment process. Indeed, cancer cells have been shown to undergo metabolic adaptations during the dissemination phase of metastasis, indicating that metabolism adapts after the loss of anchorage [21]. Many metabolites that were observed in the trypsinized samples were related to energy utilization (e.g., fatty acids and lactate). Given that metabolism can respond to stressors within seconds [22], it is highly possible that the trypsinization itself can greatly shift the metabotype of cells. Further investigation is needed to understand if these metabolic differences are a consequence of the technique, or if they are part of the biological response to matrix detachment. In contrast, the scraping method allows for the immediate quenching of metabolism with the addition of a cold organic solution directly onto adhered cells, minimizing any metabolic adaptations to occur during the detachment process. This information is of great importance as our data suggest that the metabolic response to these two detachment methods are significantly different. Therefore, not only should researchers consider the implications of these detachment methods on the extraction efficiencies of metabolites, but they should also consider that the metabolism of cells can significantly shift in response to these conditions (such as trypsinization) in a relatively small timeframe, which may change the interpretability of the study results. It should also be noted that differences in matrices, particularly between trypsinized and scraped samples, were minimized through the dilution and washing of trypsinized samples prior to metabolite extraction. The use of a high-organic solution for metabolite extraction (80% methanol) also served to precipitate any remaining trypsin prior to analysis, further diminishing potential matrix effects.

In contrast to the detachment method, the two lysis methods overall had more subtle differences in metabolic profiles, although they were still distinct in the multivariate analysis. For example, some cholesterols, acylcarnitines, and polyamines (spermine and spermidine) showed large differences between the freeze–thaw and homogenizer bead methods. Although this is a minor effect overall compared to the detachment methods, care should be taken in selecting the best lysis method based on the needs of the study being performed. Using pathway analysis, we were able to find differences in certain metabolic pathways even if certain individual metabolites did not seem to be significantly affected. From this, we were able to see changes at a broader level across the different sample preparation methods. This analysis also showed that the differences between the two detachment methods are much broader than what was seen between the lysis methods (sixteen perturbed pathways vs. four perturbed pathways). Careful consideration should be taken to ensure the best sample preparation method is used depending on the metabolic pathways of interest, particularly those that were identified as significant in our pathway analysis. While other studies have investigated the effects of trypsinization on metabolite classes, ours is the first to show a significant effect on acylcarnitines that were elevated following trypsin. This may be due to a specific property of MDA-MB-231 cells, or it could be that other studies did not include acylcarnitines in their metabolomics libraries. Regardless, this poses questions about the role of fatty acid metabolism in response to trypsin or other proteases. Cancer cells are known to undergo metabolic adaptations following matrix detachment, particularly glucose and glutamine metabolism, as a method to combat anoikis and oxidative stress during metastatic processes [21,23]. It is possible that the significant changes in acylcarnitines also indicate that alterations in fatty acid occur during the detachment process, either as a method to supply alternate energy sources or as a method to create antiapoptotic signals to survive the detachment process. Lastly, our findings that many metabolites significantly increase or decrease across these sample preparation methods was also reflected in the entire peak set (Figure 4). This indicates that there are many unknown metabolites, beyond those identified in this manuscript, that are heavily affected by these detachment and lysis methods. This indicates the need for additional studies to further identify this metabolomics “dark matter” to better understand the metabolite classes and pathways affected by these processes.

## 4. Materials and Methods

### 4.1. Chemical and Reagents

All solvents for UHPLC–HRMS analysis (Optima-grade water and methanol with 0.1% formic acid) and fetal bovine serum (FBS) were purchased from Fisher Scientific (Waltham, MA, USA). Dulbecco’s Modified Eagle Medium (DMEM) high glucose and phosphate-buffered saline were purchased from Gibco (Grand Island, NY, USA). Reagents for the bicinchoninic acid (BCA) assays were purchased from Thermo Scientific (Madison, WI, USA). The MDA-MB-231 cell line was purchased from the American Type Culture Collection (ATCC) (Manassas, VA, USA). MagNA lyser tubes with ceramic beads were purchased from Roche Diagnostics (Indianapolis, IN, USA).

### 4.2. Cell Culture

MDA-MB-231 cells were maintained in DMEM high glucose supplemented with 10% FBS, 2 mM glu- tamine, 50 U/mL penicillin, and 50 μg/mL streptomycin. Cells were grown in a 37 °C, 5% CO_2_, humidity-controlled environment. To prepare cells for different extraction methods, 4 × 10^6^ MDA-MB-231 cells were plated into 100 mm tissue culture-treated dishes and allowed to adhere overnight, achieving approximately 80% confluency. Blank culture dishes were used for all of the following methods to generate method blanks.

### 4.3. Cell Scraping Detachment Method

Culture dishes containing MDA-MB-231 cells were placed on ice. Media was aspirated and dishes were washed with 5 mL of cold PBS. After removing the PBS, 2 mL of ice-cold Homogenization Solution (80–20 methanol-water) was added to each dish for metabolite quenching. Cells were lifted off culture dishes by the use of cell scrapers. For each dish, 500 µL of the cell suspension was aliquoted into a tube for freeze–thaw lysis and another 500 µL was aliquoted into a tube for bead homogenization.

### 4.4. Trypsinization Detachment Method

To detach cells by trypsinization, the media were aspirated, and dishes were washed with 5 mL of cold PBS. After removal of PBS, 1 mL of Trypsin-EDTA (0.25%) was added to each dish followed by incubation at 37 °C for 5 min. Trypsin-EDTA was then neutralized by the addition of 10 mL DMEM, which was then removed by aspirating the supernatant after centrifuging cell suspensions for 5 min at 0.9 rpm at 8 °C. Cell pellets were washed with 2 mL ice-cold PBS and then re-centrifuged using the above parameters to aspirate the PBS. Cell pellets were then resuspended in 2 mL ice-cold Homogenization Solution. For each cell suspension, 500 µL was aliquoted into a tube for freeze–thaw lysis and another 500 µL was aliquoted into a tube for bead homogenization.

### 4.5. Bead Homogenization

As described above, 500 µL aliquots of cell suspensions from both the cell scraping and trypsinization detachment methods were placed in MagNa lyser tubes with pre-washed ceramic beads. Tubes were placed onto an Omni Bead Ruptor Elite (OMNI International) at 6.00 m/s for 2 cycles at 45 s each with a 30 s dwell time in between. Lysates were immediately placed on ice following the homogenization procedure.

### 4.6. Freeze–Thaw Cycling

As described above, 500 µL aliquots of cell suspensions from both detachment methods were placed into tubes for freeze–thaw cycling. Tubes were placed into liquid nitrogen for 30 s for freezing and then placed into a hot water bath set to 37 °C for 30 s to thaw. This process was completed a total of three times to facilitate cell lysis. Lysates were immediately placed on ice following the freeze–thaw procedure.

### 4.7. Protein Measurement

Aliquots of 100 µL from each sample were dried by speedvac for 2 h and then redissolved in 400 µL of a 5% sodium dodecyl sulfate (SDS) solution. Protein concentrations for each sample were measured by a BCA assay. Additional Homogenization Solution was added to the remaining 400 µL of each sample to normalize the metabolites by protein concentration.

### 4.8. UHPLC–HRMS Data Acquisition and Multivariate Statistical Analysis

Prior to analysis, a total quality control study pool (QCSP) was generated by combining 10 µL of each sample. Individual study samples, blanks (LC-MS grade water), and the QCSP were vortexed at 5000 rpm for 10 min using a multi-tube vortex. Samples were centrifuged at 16,000 rcf for 10 min at 4 °C, transferred to autosampler vials, and 5 µL was injected for analysis. The individual study samples were randomized, and blanks and QCSP injections were interspersed amongst the study sample in the run sequence. Data were acquired using a Vanquish UHPLC system coupled to a Q Exactive™ HF-X Hybrid Quadrupole-Orbitrap Mass Spectrometer (Thermo Fisher Scientific, San Jose, CA, USA) using previously published methods [24,25,26,27]. Metabolites were separated via an HSS T3 C18 column (2.1 × 100 mm, 1.7 µm, Waters Corporation) at 50 °C with a binary mobile phase of water (A) and methanol (B), each containing 0.1% formic acid (*v*/*v*). The UHPLC linear gradient started from 2% B, and increased to 100% B in 16 min, then held for 4 min, with the flow rate at 400 µL/min. Data-dependent acquisition of the untargeted data was acquired from 70 to 1050 *m/z* in positive mode. Peak picking, alignment, and normalization was performed using Progenesis QI (version 2.1, Waters Corporation). Peaks that had a higher average abundance in the blanks as compared to the QCSP were filtered out of the dataset. Principal Component Analysis (PCA) and Orthogonal Partial Least Squares-Discriminant Analysis (OPLS-DA) was performed on the normalized, preprocessed data using SIMCA 16 (Sartorius Stedim Data Analytics AB, Umeå, Sweden). Unit Variance (UV) scaling was used for all multivariate plots.

### 4.9. Pathway Analysis

Pathway analysis was performed using MetaboAnalyst 5.0 [28] to identify metabolic pathways that were significantly altered across the sample preparation methods. All peaks from the normalized, preprocessed data (4479) were input into the “Functional Analysis” module as a peak intensity table and grouped based upon their detachment and lysis method. A mass tolerance of 5 ppm was used, and the retention time information was included for each peak. Pathway significance was calculated using the default top 10% of peaks by *p*-value (ANOVA, calculated by MetaboAnalyst) with the Homo sapiens (human) [MFN] option selected as the pathway library. Both the mummichog and GSEA algorithms were selected to generate pathway scatter plots. Additionally, a heatmap of all peaks, across all groups, was generated using the “Functional Analysis” module of MetaboAnalyst to compare peak abundance profiles across each extraction method.

### 4.10. Metabolite Identification

Metabolites were identified in Progenesis QI using an in-house physical standards library of reference standards. All reference standards were analyzed under the same conditions used to analyze the study samples. Peaks were matched to metabolites in the in-house library by exact mass (MS), fragmentation pattern (MS/MS), and retention time (RT). An ontology system was provided for each peak match to indicate the evidence basis for each identification. A peak was considered to have a match by RT if the peak eluted within 0.5 min compared to the reference standard; an MS match was defined as <5 ppm error compared to the theoretical mass based on the metabolite chemical formula; and an MS/MS match was defined as a similarity score > 30 to a reference standard (calculated using MS/MS match algorithms in Progenesis QI). OL1 refers to an in-house metabolite match by RT, MS, and MS/MS; OL2a refers to a match by RT and MS only; and OL2b refers to a match by MS and MS/MS only.

### 4.11. Statistical Analysis of Identified Metabolites

Metabolites that were matched to the in-house library by an OL1, OL2a, or OL2b definition were input into the “Statistical Analysis” module in MetaboAnalyst as an intensity table using abundance values from the normalized, preprocessed dataset. Heatmaps and One-way Analysis of Variance (ANOVA) modules in MetaboAnalyst were used to determine the changes in these metabolites across the detachment and lysis methods.

## 5. Conclusions

In conclusion, detachment and lysis methods produce significantly different metabolic profiles (in terms of the number of features detected, and their abundance) in MDA-MB-231 cells. Overall, scraped samples had a higher abundance of metabolites although it should be noted that a subset of metabolites increased in trypsinized samples. These increased metabolites may be due to enhanced yield, or they may be a consequence of the biological response to matrix detachment. Interestingly, this is an observation that was seen in another study using HeLa and MCF-7 cells [20]. As such, great care must be taken when selecting trypsinization as a detachment method for metabolomics studies, as some of the metabolic changes may be influenced by the acute metabolic response to proteolytic release from the extracellular matrix, such as fatty acid metabolism, as seen by the significant increases in acylcarnitines in trypsinized samples. The difference between lysis methods was less pronounced, with both freeze–thaw cycling and bead homogenization producing similar results for most metabolites. These results provide a better understanding for how different sample preparation procedures affect extracted metabolite profiles, which will aid in future studies investigating the metabolism of triple-negative breast cancer cells. Furthermore, this study shows how MDA-MB-231 cells—a highly common model for triple-negative breast cancer—responds to various commonly used sample preparation methods. Future studies are needed to determine if these trends are also observed in other cell models.

## Figures and Tables

**Table 1:** Significance values of the perturbed pathways due to detachment and lysis methods.

Trypsinized vs. Scraped			
Pathway Name	Mummichog_Pvals	GSEA_Pvals	Combined_Pvals
Tyrosine metabolism	0.00071	0.0101	9.00 × 10^-5^
Urea cycle/amino group metabolism	0.00285	0.01075	0.00035
Arginine and proline metabolism	0.00153	0.02273	0.00039
Vitamin B6 (pyridoxine) metabolism	0.00845	0.01282	0.0011
Tryptophan metabolism	0.02778	0.01053	0.00267
Aspartate and asparagine metabolism	0.01479	0.03061	0.00394
Vitamin B3 (nicotinate and nicotinamide) metabolism	0.02075	0.05952	0.00951
Starch and sucrose metabolism	0.06117	0.02326	0.01075
Methionine and cysteine metabolism	0.03379	0.04494	0.01137
Glycine, serine, alanine and threonine metabolism	0.1673	0.01087	0.0133
Alanine and aspartate metabolism	0.03482	0.06098	0.01519
Nitrogen metabolism	0.06117	0.04651	0.01952
Nucleotide sugar metabolism	0.2055	0.01852	0.02501
Benzoate degradation via CoA ligation	0.04187	0.1176	0.03109
N-Glycan biosynthesis	0.5143	0.0119	0.03731
Dynorphin metabolism	0.2055	0.04167	0.04933
**Homogenizer Beads vs. Freeze–Thaw**			
**Pathway Name**	**Mummichog_Pvals**	**GSEA_Pvals**	**Combined_Pvals**
Tryptophan metabolism	0.00656	0.05263	0.0031
De novo fatty acid biosynthesis	0.04632	0.01818	0.0068
Fatty acid activation	0.02136	0.08621	0.01344
Fatty acid metabolism	0.02136	0.1552	0.02224

**Table 2:** Average intensities and relative standard deviations for the identified peaks.

	Scrape–Freeze–Thaw		Scrape–Homogenize		Trypsinize–Homogenize		Trypsinize–Freeze–Thaw	
Compound	Avg	RSD (%)	Avg	RSD (%)	Avg	RSD (%)	Avg	RSD (%)
Putrescine_OL1_0.54_89.1075 *m/z*	2992	42.4	1674	86.0	1014	44.9	1525	96.9
L-Lysine_OL1_0.56_147.1130 *m/z*	22,207	31.3	20,342	38.4	5156	27.9	6412	8.5
Spermidine_OL1_0.59_146.1654 *m/z*	202,200	27.4	41,381	52.4	367,082	59.1	28,400	82.2
Spermine_OL1_0.65_203.2233 *m/z*	110,891	51.5	25,909	52.8	271,520	69.6	22,128	55.8
Histidine_OL1_0.67_155.0698 *n*	168,403	9.0	163,603	12.7	32,176	17.6	31,296	32.0
Arginine_OL1_0.67_175.1192 *m/z*	265,818	11.6	267,533	20.4	33,540	29.1	27,417	61.0
N,N,N-Trimethyllysine_OL1_0.67_189.1601 *m/z*	6356	36.9	6554	12.1	3118	6.8	2329	86.7
Glutamine_OL1_0.69_146.0694 *n*	1,472,545	8.0	1,442,283	2.4	704,975	17.0	761,455	39.8
Thiamine_OL1_0.69_265.1120 *m/z*	305,026	7.0	282,389	12.7	56,499	12.8	68,323	86.7
Creatinine_OL1_0.71_113.0591 *n*	175,014	7.3	165,813	16.8	92,045	14.8	114,427	19.5
Threonine_OL1_0.71_120.0657 *m/z*	77,169	4.9	76,222	6.0	51,248	19.8	53,769	41.3
L-Cartinine_OL1_0.71_162.1127 *m/z*	526,264	13.4	475,538	19.5	396,120	20.1	421,175	80.6
Galactitol_OL1_0.71_205.0686 *m/z*	237,325	8.5	230,700	9.0	112,259	5.8	102,267	59.3
Lactose_OL1_0.71_342.1166 *n*	230,068	25.0	206,361	12.1	1,776,784	10.5	2,207,958	18.9
Creatine_OL1_0.72_132.0770 *m/z*	94,761	8.1	94,497	8.2	143,864	5.9	125,815	83.2
Deoxycarnitine_OL1_0.72_146.1178 *m/z*	20,921	26.2	20,727	18.6	34,590	33.9	22,083	97.2
L-Proline_OL1_0.75_115.0635 *n*	351,132	9.3	341,882	14.4	470,374	31.4	623,017	31.3
L-Serine_OL1_0.78_105.0428 *n*	43,852	5.8	43,025	8.9	22,294	6.9	22,194	30.6
L-Asparagine_OL1_0.8_133.0610 *m/z*	9458	10.3	8823	7.9	7919	18.2	7657	55.1
2-Aminoadipic Acid_OL1_0.8_162.0763 *m/z*	103,200	9.2	100,713	4.3	60,123	5.9	80,185	17.9
S-Adenosylmethionine_OL1_0.8_399.1450 *m/z*	27,144	23.1	22,708	32.7	44,605	31.2	65,449	16.4
L-Glutamic Acid_OL1_0.82_147.0534 *n*	2,140,291	9.1	2,015,112	7.4	874,729	10.2	990,198	18.5
N-Acetylserine_OL1_0.87_170.0427 *m/z*	385,191	11.3	339,478	11.8	433,571	10.8	535,675	9.8
1-Aminocyclopropanecarboxylic Acid_OL1_0.91_101.0479 *n*	539,979	8.6	502,411	8.6	446,443	7.9	495,069	16.7
D-Aspartic Acid_OL1_0.91_134.0450 *m/z*	671,296	18.1	594,642	17.2	828,733	11.0	945,140	19.8
L-Cystine_OL1_0.91_241.0316 *m/z*	104,297	12.3	100,058	18.5	2009	75.5	1402	35.1
L-Serine_OL1_0.91_88.0395 *m/z*	19,781	14.6	18,576	15.6	23,792	9.8	27,695	17.2
Phosphorylcholine_OL1_0.98_183.0664 *n*	18,637,731	8.7	17,187,394	14.4	18,140,958	11.8	26,579,422	10.6
Acetylcarnitine_OL1_1.07_204.1235 *m/z*	33,374	13.8	29,443	22.0	37,426	11.6	48,813	17.9
Nicotinamide_OL1_1.16_123.0555 *m/z*	408,693	16.8	400,555	14.2	54,688	1.2	57,120	7.4
N-Acetylaspartate_OL1_1.27_175.0484 *n*	80,089	9.7	77,520	12.5	78,246	10.4	101,863	13.7
L-Glutathione Reduced_OL1_1.32_307.0843 *n*	24,688,792	14.7	21,418,891	19.9	30,277,732	8.3	41,337,083	11.9
Pyridoxine_OL1_1.36_169.0741 *n*	1,102,166	13.7	977,926	13.8	30,102	11.4	39,539	12.4
Pyroglutamic Acid_OL1_1.49_130.0502 *m/z*	2,123,041	12.2	2,054,021	12.5	354,201	7.6	395,546	16.4
Succinic Acid_OL1_1.67_118.0268 *n*	39,389	1.1	44,925	16.5	24,518	20.4	23,208	10.2
N-Acetylglutamate_OL1_1.76_189.0641 *n*	27,615	8.1	26,479	14.4	26,836	3.6	31,166	11.2
Leucine_OL1_1.87_131.0949 *n*	17,790,828	11.3	16,285,136	9.7	2,683,726	9.5	3,369,993	12.6
Glycodeoxycholic Acid_OL1_13.07_449.3147 *n*	31,623	26.9	28,427	18.5	4	173.2	488	3.1
Palmitoleic Acid_OL1_13.6_254.2250 *n*	45,654	4.2	48,022	4.4	49,662	5.6	47,474	10.8
Propanoylcarnitine_OL1_2.17_218.1391 *m/z*	143,866	12.8	128,195	16.4	108,344	13.1	147,557	14.2
Adenosine_OL1_2.62_268.1045 *m/z*	322,597	14.4	234,125	23.3	157,091	65.4	64,987	16.1
Kynurenine_OL1_3.09_209.0926 *m/z*	8736	13.0	7779	18.4	1318	15.1	1321	69.8
L-Phenylalanine_OL1_3.4_165.0793 *n*	148,133	8.3	127,736	8.3	21,789	11.4	26,215	18.3
Butanoylcarnitine_OL1_3.7_232.1549 *m/z*	65,075	15.5	62,277	3.8	326,341	17.7	424,502	18.7
Pantothenate_OL1_4.16_219.1112 *n*	2,002,286	5.7	1,819,190	15.1	1,150,498	10.8	1,601,215	13.8
Valerylcarnitine_OL1_4.86_246.1705 *m/z*	87,542	11.4	76,800	19.6	43,760	17.4	58,454	19.0
Methylthioadenosine_OL1_4.95_298.0974 *m/z*	247,549	7.6	247,579	11.7	203,744	19.4	220,645	13.0
4-Hydroxybenzaldehyde_OL1_5.44_105.0337 *m/z*	3808	23.8	3294	12.9	567	1.8	559	7.5
2-Hydroxyphenylacetate_OL1_6.47_153.0549 *m/z*	12,314	4.1	13,598	9.1	11,840	4.3	11,954	14.8
Riboflavin_OL1_6.74_377.1463 *m/z*	45,334	21.5	36,882	24.1	590	27.5	1343	24.9
2-Octenedioic Acid_OL1_7.13_173.0811 *m/z*	5113	44.6	7319	57.7	2395	51.0	4038	92.4
Azelate_OL1_8.7_188.1053 *n*	268,336	2.9	253,718	4.8	253,716	2.9	288,027	12.8
Lumichrome_OL1_8.72_265.0701 *m/z*	3870	22.1	4448	14.0	2179	26.4	1606	89.5
Glycerol_OL2a_0.6_115.0367 *m/z*	1121	32.7	2066	10.9	1477	31.3	2534	50.0
Creatine_OL2a_0.63_263.1465 *m/z*	75,190	21.2	70,905	20.0	1227	113.3	2808	59.6
N,N,N-Trimethyllysine_OL2a_0.65_211.1420 *m/z*	577	123.1	287	173.2	0	-	373	90.5
Histidine_OL2a_0.67_178.0590 *m/z*	12,284	7.7	13,639	8.6	3812	31.7	3235	60.2
Sarcosine_OL2a_0.69_112.0370 *m/z*	868	32.3	742	27.9	1370	7.2	1159	81.7
L-Lysine_OL2a_0.69_129.1024 *m/z*	598	72.2	181	173.2	603	84.7	2139	36.0
Betaine_OL2a_0.69_150.1127 *m/z*	0	-	1523	142.4	315	97.1	99	122.0
Betaine_OL2a_0.71_140.0684 *m/z*	104,380	9.4	94,252	13.8	16,865	21.0	21,989	21.6
Galactose_OL2a_0.71_180.0637 *n*	9,339,956	10.7	8,638,770	7.4	2,165,989	18.2	2,210,381	14.0
Galactose_OL2a_0.71_219.0269 *m/z*	888,396	7.0	783,629	6.6	376,622	18.2	529,905	20.4
Trimethylamine Oxide_OL2a_0.71_76.0758 *m/z*	624	52.7	815	32.0	105	62.9	31	173.2
Arabitol_OL2a_0.72_175.0580 *m/z*	8611	47.2	8464	15.0	3476	13.8	2267	86.6
Sorbitol_OL2a_0.72_221.0425 *m/z*	14,185	8.6	13,373	2.7	6125	21.6	6555	80.8
Sucrose_OL2a_0.72_325.1133 *m/z*	520	173.2	420	173.2	6975	17.8	4244	89.9
Pipecolate_OL2a_0.72_94.0653 *m/z*	3490	27.8	5576	16.4	6494	53.1	1542	57.2
Uracil_OL2a_0.75_113.0347 *m/z*	3350	30.5	3567	9.9	6473	53.9	3620	13.5
N-Acetylputrescine_OL2a_0.75_113.1075 *m/z*	0	-	160	173.2	607	70.5	21	173.2
Glycerol_OL2a_0.75_115.0368 *m/z*	6206	50.0	6747	12.6	1357	32.5	1147	93.2
Creatine_OL2a_0.75_154.0589 *m/z*	3381	48.7	4280	19.9	3520	4.8	2818	87.7
Deoxyribose_OL2a_0.76_117.0548 *m/z*	11,747	15.8	10,872	43.0	4871	14.0	5724	63.0
Choline_OL2a_0.76_143.0705 *m/z*	3740	10.7	3714	7.9	4828	12.2	3992	17.7
Taurine_OL2a_0.78_126.0221 *m/z*	7535	30.2	7801	24.1	68,493	22.3	71,492	38.9
3-(Carbamoylamino)Propanoic Acid_OL2a_0.78_155.0429 *m/z*	4671	28.5	4045	43.0	7551	2.6	6199	65.8
Pyridoxal_OL2a_0.78_168.0658 *m/z*	1977	18.9	2218	36.2	917	14.3	518	72.0
Fructose_OL2a_0.78_213.0973 *m/z*	181	87.8	59	173.2	3964	15.8	3376	87.2
Glycine_OL2a_0.78_76.0394 *m/z*	16,396	6.1	15,563	7.8	17,457	11.3	18,217	31.4
5-Aminolevulinate_OL2a_0.8_132.0657 *m/z*	7413	13.8	8389	15.6	7112	10.4	6998	15.0
3-Methyladenine_OL2a_0.8_150.0777 *m/z*	1058	13.8	1531	7.9	11,761	78.6	1813	30.9
Spermine_OL2a_0.8_203.2234 *m/z*	71,359	30.7	41,512	20.4	183,518	57.7	11,527	48.6
Deoxyribose_OL2a_0.8_99.0442 *m/z*	13,993	4.7	13,684	5.7	16,206	2.2	16,056	6.2
Xylose_OL2a_0.82_115.0392 *m/z*	7632	15.1	7779	7.4	9921	11.4	10,416	16.3
5-Aminopentanoate_OL2a_0.82_118.0865 *m/z*	722,647	13.1	669,054	13.3	187,580	3.1	193,015	31.4
Glutamyl-Valine_OL2a_0.82_247.1292 *m/z*	20,682	27.9	18,403	19.8	70	173.2	53	173.2
L-Lysine_OL2a_0.91_147.1131 *m/z*	87,495	16.5	78,912	15.1	7815	6.9	8954	17.9
Dihydroorotic Acid_OL2a_0.91_191.0666 *m/z*	7007	15.9	6597	37.0	7747	11.6	5671	47.1
Cytidine_OL2a_0.91_276.1194 *m/z*	136,179	23.1	126,828	18.5	16,521	10.8	19,062	15.1
Hypotaurine_OL2a_0.96_110.0272 *m/z*	2926	14.9	2486	13.9	2760	15.6	3432	23.4
Phosphorylcholine_OL2a_0.96_183.0664 *n*	162,563	29.0	132,261	47.2	135,874	40.8	391,675	37.4
Betaine_OL2a_1.05_118.0865 *m/z*	1,157,973	9.1	1,053,217	12.3	268,966	3.9	311,624	6.2
Pyridoxal_OL2a_1.14_168.0658 *m/z*	3417	15.3	3279	22.2	763	21.0	643	55.8
L-Glutathione Reduced_OL2a_1.14_290.0808 *m/z*	1975	49.5	1679	49.3	2154	16.0	6728	92.6
Shikimic Acid_OL2a_1.16_139.0392 *m/z*	1048	16.7	1247	6.5	887	26.9	1004	22.1
L-Methionine_OL2a_1.18_172.0406 *m/z*	8726	14.2	6260	22.7	173	87.8	396	57.8
Uric Acid_OL2a_1.27_169.0359 *m/z*	5373	18.1	4778	25.6	0	-	0	-
N-Acetylaspartate_OL2a_1.29_158.0451 *m/z*	1920	14.8	1627	35.3	2542	14.3	2536	60.6
Xanthine_OL2a_1.65_153.0410 *m/z*	12,029	19.1	10,254	6.7	165	173.2	0	-
L-Tyrosine_OL2a_1.71_204.0635 *m/z*	23,973	19.5	20,174	8.0	1793	19.2	2099	18.7
Sebacate_OL2a_10.06_225.1102 *m/z*	92,992	5.6	92,015	3.1	92,176	2.8	103,904	4.8
2-(2-Carboxyethyl)-4-Methyl-5-Propylfuran-3-Carboxylic Acid (CMPF)_OL2a_11.97_263.0895 *m/z*	3580	12.5	3631	11.5	3347	12.9	3506	6.4
12-Hydroxydodecanoic Acid_OL2a_12.05_239.1622 *m/z*	300,056	5.7	276,373	2.7	266,376	3.2	326,278	0.1
17-Octadecynoic Acid_OL2a_14.74_303.2296 *m/z*	3451	17.0	3669	23.0	3747	10.7	4109	16.9
Oleic Acid_OL2a_15.49_283.2634 *m/z*	4171	39.5	9314	5.9	12,433	23.0	5478	6.8
Oleic Acid_OL2a_15.67_283.2635 *m/z*	716	106.3	2885	35.4	2845	44.9	538	87.0
25-Hydroxycholesterol_OL2a_15.86_367.3361 *m/z*	5383	28.5	31,189	14.6	21,320	21.8	15,555	31.2
Arachidonic Acid_OL2a_15.88_287.2371 *m/z*	0	-	692	97.0	3528	43.2	1462	46.3
24-Hydroxychloesterol_OL2a_16.12_425.3393 *m/z*	2979	51.4	6453	25.3	8977	14.7	12,584	20.6
10Z-Nonadecenoic Acid_OL2a_16.23_319.2610 *m/z*	1139	119.5	1296	72.6	6095	12.4	5706	15.5
10Z-Nonadecenoic Acid_OL2a_16.25_297.2792 *m/z*	895	117.9	206	173.2	3779	21.6	2407	32.8
7-Dehydrocholesterol_OL2a_17.29_407.3288 *m/z*	2855	22.6	1802	35.4	9337	27.6	11,165	1.8
5-Hydroxytryptophan_OL2a_2.5_185.0708 *m/z*	2510	16.1	1877	15.5	2485	16.8	3953	8.6
4-Acetamidobutanoic Acid_OL2a_2.59_168.0636 *m/z*	13,487	12.8	10,492	11.3	847	51.9	998	83.1
Cyclic AMP_OL2a_3.04_330.0603 *m/z*	755	39.1	914	64.0	10,860	3.2	8639	69.4
Methyglutarate_OL2a_4.49_169.0475 *m/z*	11,990	5.5	11,958	6.8	11,634	4.9	11,874	13.4
L-Tryptophan_OL2a_4.49_227.0796 *m/z*	45,154	14.4	38,462	10.7	7886	12.8	8910	30.9
Tiglylcarnitine_OL2a_4.59_244.1548 *m/z*	6477	4.4	5385	13.6	4495	3.9	3982	86.8
3-Propylmalic Acid_OL2a_4.78_176.0688 *n*	8328	20.2	46,028	22.2	65,600	19.5	9590	11.2
3-Hydroxysuberic Acid_OL2a_5.2_173.0812 *m/z*	1098	61.7	761	54.0	337	56.2	362	51.2
Hippuric Acid_OL2a_5.44_202.0478 *m/z*	20,814	18.4	18,500	13.1	2256	31.7	2030	10.9
3,3-Dimethylpentanedionate_OL2a_6.08_183.0632 *m/z*	26,837	7.0	25,321	8.2	22,685	1.8	26,993	7.0
N-Acetylleucine_OL2a_6.25_196.0949 *m/z*	1666	33.7	2598	42.8	877	36.9	1382	48.1
2-Hydroxyphenylacetate_OL2a_6.32_135.0442 *m/z*	7126	10.8	8264	7.3	8772	4.1	8172	2.9
Phenylethanolamine_OL2a_6.81_120.0810 *m/z*	844	33.1	1348	41.7	1730	33.0	1437	28.2
2-Octenedioic Acid_OL2a_6.81_195.0632 *m/z*	3387	13.2	2517	12.6	2645	16.8	2972	15.3
Dethiobiotin_OL2a_7.37_429.2716 *m/z*	201	173.2	229	87.2	59,537	22.1	78,517	17.8
2-Octenedioic Acid_OL2a_7.42_195.0631 *m/z*	7730	17.3	5094	9.2	3140	38.0	7516	8.4
Suberate_OL2a_7.47_197.0789 *m/z*	129,165	4.3	118,707	4.1	117,587	2.1	137,784	4.8
Suberate_OL2a_7.6_157.0862 *m/z*	23,101	11.6	23,341	6.1	19,390	8.1	20,297	13.2
Caprylate_OL2a_9.32_167.1047 *m/z*	27,549	5.2	27,692	7.5	28,494	2.5	32,939	3.8
Sebacate_OL2a_9.72_225.1102 *m/z*	52,739	6.7	48,714	3.8	46,481	12.6	53,830	6.9
10-Hydroxy-2-Decenoic Acid_OL2a_9.84_209.1152 *m/z*	117,259	4.7	116,924	2.7	115,816	4.8	129,228	0.7
3-Hydroxyadipic 3,6 Lactone_OL2b_0.69_144.0424 *n*	34,905	7.9	31,933	9.1	56,260	19.5	64,756	13.0
Acetoacetate_OL2b_0.71_102.0318 *n*	39,375	8.5	36,703	2.3	37,028	3.1	40,002	8.9
3-Hydroxy-3-Methylglutaric Acid_OL2b_0.75_185.0424 *m/z*	17,639	20.2	15,247	11.9	3647	10.1	3117	19.3
Propionyl-L-Carnitine_OL2b_0.76_218.1391 *m/z*	240,975	7.0	229,428	9.6	216,814	11.9	354,568	18.6
Adipate_OL2b_0.8_111.0442 *m/z*	34,128	1.6	34,202	5.8	33,653	4.9	35,715	0.7
Pyridoxine_OL2b_0.8_169.0741 *n*	286,515	17.4	256,692	9.7	15,455	13.7	14,008	71.9
Pyroglutamic Acid_OL2b_0.82_259.0929 *m/z*	11,248	38.6	10,178	24.9	0	-	0	-
2-Hydroxybutyrate_OL2b_0.82_87.0442 *m/z*	8654	7.0	8841	5.6	10,005	4.0	10,868	0.3
L-Cysteinylglycine_OL2b_1.29_179.0488 *m/z*	157,188	14.9	137,522	22.2	194,013	8.6	266,356	10.1
Glutarate_OL2b_1.67_115.0392 *m/z*	29,496	2.4	30,845	5.5	26,960	9.6	26,663	9.5
3-Methyladenine_OL2b_1.87_150.0777 *m/z*	768	22.7	429	136.3	2705	66.5	1279	11.0
Benzoate_OL2b_10.34_105.0337 *m/z*	1707	5.5	1842	17.8	1346	28.5	1501	48.1
2-Octenedioic Acid_OL2b_11.17_173.0811 *m/z*	0	-	0	-	1342	99.6	0	-
10-Hydroxydecanoic Acid_OL2b_11.39_153.1277 *m/z*	9020	1.7	8179	0.7	8623	8.7	8677	7.6
10-Hydroxydecanoic Acid_OL2b_11.42_171.1383 *m/z*	11,427	0.8	10,545	5.9	10,166	7.9	10,899	8.0
Sphinganine_OL2b_11.92_302.3058 *m/z*	54,577	7.3	55,845	2.8	53,097	6.7	57,716	2.4
Azelate_OL2b_12.05_153.0912 *m/z*	6061	7.5	6831	2.1	6209	14.8	6855	5.6
Heptanoic Acid_OL2b_12.45_283.1883 *m/z*	48,315	4.2	46,278	5.3	45,251	2.9	52,254	8.8
Sphinganine_OL2b_12.47_284.2952 *m/z*	2750	47.7	2659	27.2	5898	38.0	7522	50.9
2-Hydroxytetradecanoic Acid_OL2b_12.73_267.1934 *m/z*	43,587	4.9	42,755	9.2	44,157	2.7	48,037	6.1
Sphinganine_OL2b_12.75_302.3058 *m/z*	16,252	19.1	16,622	25.9	47,603	11.8	56,698	10.6
Sphinganine_OL2b_12.82_284.2952 *m/z*	3644	18.8	4261	20.3	13,496	17.6	13,425	15.9
Hexadecanoylcarnitine_OL2b_13.1_400.3428 *m/z*	16,550	7.3	15,958	4.4	19,620	11.5	25,724	12.2
Benzoate_OL2b_13.15_105.0337 *m/z*	1858	8.3	1680	1.0	1746	6.3	1752	10.5
Linoleic Acid_OL2b_13.19_263.2374 *m/z*	9159	13.6	8004	5.8	7610	11.6	8222	2.5
Oleoylcarnitine_OL2b_13.27_426.3584 *m/z*	20,182	12.4	18,244	24.1	17,383	14.8	24,494	10.1
12-Hydroxy-9-Cis-Octadecenoic Acid_OL2b_13.47_263.2373 *m/z*	11,675	12.9	10,852	8.4	7759	5.0	8027	8.6
Octadecanoylcarnitine_OL2b_13.67_428.3741 *m/z*	22,954	10.3	22,440	12.7	26,152	20.7	34,914	22.5
Linoleic Acid_OL2b_13.9_263.2373 *m/z*	6141	25.8	5205	14.5	1131	9.4	1722	23.7
2-Hydroxytetradecanoic Acid_OL2b_13.92_267.1933 *m/z*	27,633	1.3	26,690	1.8	25,513	7.7	26,874	3.6
Palmitoleic Acid_OL2b_14.1_254.2249 *n*	13,604	8.8	16,263	5.6	16,053	11.1	15,906	10.2
12-Hydroxy-9-Cis-Octadecenoic Acid_OL2b_14.28_321.2403 *m/z*	494,789	4.8	560,842	2.0	561,252	4.2	532,630	6.3
2-Hydroxyglutarate_OL2b_14.35_297.0827 *m/z*	4411	17.7	6365	28.2	6406	18.4	4074	8.4
Palmitoleic Acid_OL2b_14.58_254.2248 *n*	46,698	14.3	51,379	12.5	58,247	14.3	57,708	8.9
Palmitoleic Acid_OL2b_14.79_254.2248 *n*	40,233	8.1	51,315	12.9	60,231	9.3	47,499	11.6
Eicosapentaenoate_OL2b_14.94_325.2140 *m/z*	867	10.3	15,576	58.1	11,425	42.1	711	10.4
Docosahexaenoate_OL2b_15.21_328.2403 *n*	112,516	20.5	329,323	43.5	292,259	38.5	66,066	1.5
27-Hydroxycholesterol_OL2b_15.37_385.3468 *m/z*	31,043	45.5	23,565	34.7	33,490	23.1	43,309	45.5
3-Hydroxy-3-Methylglutaric Acid_OL2b_3.21_145.0498 *m/z*	14,199	10.1	15,513	19.1	9555	24.4	9991	9.0
Β-Nicotinamide Mononucleotide_OL2b_3.28_335.0645 *m/z*	976,592	7.2	928,731	15.9	776,931	8.7	1,065,967	12.3
Leucine_OL2b_3.77_154.0842 *m/z*	55,154	49.7	68,733	49.4	30,573	74.7	40,142	85.4
N-Acetyl-D-Glucosamine_OL2b_3.8_204.0871 *m/z*	10,823	23.1	10,618	25.7	7804	18.7	10,025	15.1
10-Hydroxydecanoic Acid_OL2b_3.82_153.1277 *m/z*	5351	9.9	5687	21.9	6232	17.6	6492	9.0
Glutarate_OL2b_3.89_155.0317 *m/z*	5471	11.0	15,289	17.5	9519	18.6	2705	19.0
N-Acetylserotonin_OL2b_5.93_219.1133 *m/z*	8978	6.6	9545	10.2	8470	10.4	8677	18.3
2′,4′-Dihydroxyacetophenone_OL2b_6.18_153.0549 *m/z*	15,564	4.1	15,019	7.5	14,038	2.1	16,217	4.9
Sebacate_OL2b_6.57_167.1070 *m/z*	9903	8.2	9679	16.8	8116	19.3	8824	10.8
Sebacate_OL2b_6.81_167.1070 *m/z*	3801	13.2	3013	15.0	2798	32.3	3462	11.0
Pantothenate_OL2b_6.98_242.1004 *m/z*	4065	21.2	3328	18.3	3597	9.3	3750	10.9
Sebacate_OL2b_7.47_203.1282 *m/z*	10,163	5.2	9709	4.2	9555	4.1	10,235	9.0
Hexanoyl Glycine_OL2b_7.99_196.0948 *m/z*	35	173.2	1665	108.1	4102	59.9	116	16.6
Benzoate_OL2b_8.62_105.0337 *m/z*	1728	11.9	1440	21.0	1131	34.3	1630	9.7
Paraxanthine_OL2b_8.65_180.0641 *n*	94,367	14.5	127,026	18.4	113,606	7.2	85,535	9.1
10-Hydroxydecanoic Acid_OL2b_9.97_153.1277m/z	7148	2.5	7353	15.6	6869	1.4	7221	4.1

Metabolites are displayed in the following format: compound name_ontology level_retention time_mass. Masses ending with *n* are neutral masses whereas masses ending in *m/z* are ion masses.

## Data Availability

The data presented in this study are available on request from the corresponding author due to privacy restrictions.

## Supplementary Materials

The following supporting information can be downloaded at: https://www.mdpi.com/article/10.3390/metabo12020168/s1, Supplemental Table S1. Metabolite statistics across QC pool injections.
